# 811 Statistical Analyses of Multiple Burn Wounds within an Animal

**DOI:** 10.1093/jbcr/irac012.359

**Published:** 2022-03-23

**Authors:** Lonnie E Grantham, Angela R Jockheck-Clark, Laura L Scott

**Affiliations:** United States Army Institute of Surgical Research, Fort Sam Houston, Texas; United States Army Institute of Surgical Research, Ft Sam Houston, Texas; United States Army Institute of Surgical Research, Fort Sam Houston, Texas

## Abstract

**Introduction:**

Porcine animal models are frequently used to study the effects of various burn wound treatments and conditions. In these studies, multiple wounds are created on the dorsum of an anesthetized pig. Wounds within an animal are correlated, and the analysis of such a design should account for this dependence. Two recent studies are used as examples to illustrate the use of random effects for animal to account for the dependence of wound measurements within each pig.

**Methods:**

In both studies, treatments were randomized to wound location, and neither contained repeated measurements within a wound.

Study #1 investigated the effectiveness of enzymatic debridement. Forty 3-cm diameter partial-thickness burn wounds were created on the dorsum of 5 anesthetized pigs, arranged in 4 rows and 10 columns. Dorsal quadrants with 10 wounds each were created by combinations of two conditions: wounds that were wet or dry and wounds that were treated for either 72 or 96 hours. Debridement Efficiency Scores were calculated for each wound and evaluated for differences by inferential statistical comparisons between experimental treatments and conditions. The multilevel statistical model contained Treatment Group (5 levels), Treatment Time (2 levels: 72 hour and 96 hour), and Wet/Dry Condition (2 levels) as fixed effects, which were crossed to create a 3-way block, resulting in 2 wounds per animal for each Treatment, Wet/Dry, and Time combination. Random effects of animal and treatment block within animal were included in the model.

Study #2 investigated the effectiveness of an experimental treatment applied at 3 different post-burn injury time points versus a standard of care (SOC). Up to 12 5-cm^2^ deep partial thickness wounds were created in 2 rows and 6 columns on each dorsum of 6 anesthetized pigs. Each treated wound was compared with a SOC wound located along the same cranio-caudal axis. Paired differences for wound closure % were calculated and analyzed with time as a fixed effect with a random effect for animal.

**Results:**

For Study #1, there were 200 total wounds, and for Study #2 there were 72 total wounds. These experimental units are not independent, as required by statistical testing. For both studies, there was graphical evidence that the burn wound measurements differed both within and between pigs. The statistical tests accounted for this variability by incorporating the effects due to pigs.

**Conclusions:**

Matching the statistical model to the experimental design is critical for correct interpretation of the results. Failure to use the correct model by assuming wounds within a pig are independent (and thereby ignoring any effect due to the pig) will result in erroneous statistical test results and less powerful tests of treatment differences.

